# Wettability and Coalescence of Cu Droplets Subjected to Two-Wall Confinement

**DOI:** 10.1038/srep15190

**Published:** 2015-10-13

**Authors:** Xiongying Li, Hongru Ren, Weikang Wu, Hui Li, Long Wang, Yezeng He, Junjun Wang, Yi Zhou

**Affiliations:** 1 Key Laboratory for Liquid-Solid Structural Evolution and Processing of Materials, Ministry of Education, Shandong University, Jinan 250061, People’s Republic of China; 2 School of Material Science and Engineering, China University of Mining and Technology, Xuzhou 221116, P. R. China

## Abstract

Controlling droplet dynamics via wettability or movement at the nanoscale is a significant goal of nanotechnology. By performing molecular dynamics simulations, we study the wettability and spontaneous coalescence of Cu droplets confined in two carbon walls. We first focus on one drop in the two-wall confinement to reveal confinement effects on wettability and detaching behavior of metallic droplets. Results show that Cu droplets finally display three states: non-detachment, semi-detachment and full detachment, depending on the height of confined space. The contact angle ranges from 125° to 177°, and the contact area radius ranges from 12 to ~80 Å. The moving time of the detached droplet in the full detachment state shows a linear relationship with the height of confined space. Further investigations into two drops subjected to confinement show that the droplets, initially distant from each other, spontaneously coalesce into a larger droplet by detachment. The coalescing time and final position of the merged droplet are precisely controlled by tailoring surface structures of the carbon walls, the height of the confined space or a combination of these approaches. These findings could provide an effective method to control the droplet dynamics by confinement.

Widely recognized as one of the quintessential technologies for applications in the manipulation of science and engineering, the control of droplet dynamics, especially wettability and movement, has already been the focus of an impressive list of studies^1^,^2^,^3^,^4^,^5^. The desired wettability, typically characterized by the contact angle (CA), has been reported to be obtained by fabricating solid surfaces with micro/nanostructures^6^,^7^ or chemically modifying surfaces to decrease their surface energy^8^,^9^, which is inspired by the strategies of numerous natural phenomena, *i.e.,* self-cleaning of lotus leaves and butterfly wings, the ability of geckos to adhere to snow and their amazing climbing abilities^10^,^11^,^12^,^13^, all of which are related to the unique surface structure or surface energy. Recently, functional surfaces with a particular wettability ranging from super-hydrophobic (CA > 150°) to super-hydrophilic (CA close to 0°) have been synthesized^14^,^15^. Moreover, remarkable surfaces with reversible wettability from super-hydrophobic to super-hydrophilic have been realized^16^. In terms of droplet motions, conventional technologies to guide the printing of nanoparticles, charge jumping droplets or synthesize a nanorotor from multilayer carbon nanotubes (CNTs) are achieved by applying magnetic fields or electric current^17^,^18^,^19^. Considerable efforts have been devoted to the energy-induced spontaneous insertion of nanostructures into CNTs since their remarkable discovery^20^. These encapsulating events, however, are time consuming^21^. Recently, dewetting-induced detachment of nanodroplets, resulting from the transition of surface energy into kinetic energy^22^,^23^, has been demonstrated to provide another effective method to control droplet motions^24^,^25^. For example, Habenicht *et al.*^3^ reported that thin gold film exposed to a pulsed laser could jump from the substrate. The jumping droplet is dominated by the inertia force, which had been verified by Afkham and Kondic^23^. Furthermore, the controllable movement of metallic droplets has been emphasized by tuning temperature^22^ and regulating surface structures of solid walls^26^. The detaching droplets had been reported to possess potential applications in nanotechnology and nanodevices^27^,^28^,^29^.

Despite the thorough studies on the detaching dynamics of metallic droplets, including the detaching mechanism^3^,^23^, the conditions of detachment^30^,^31^ and controllable movements^22^,^26^, a discussion of the confinement effect on ejected droplets, as well as the investigations into possible applications of detachment, are still lacking. Confinement has been reported to be of significance in fabrications of new and promising materials. Few materials have elicited the same degree of growing research as CNTs and graphene (G) due to their excellent properties, especially their unique structures, which endow CNTs and G with the ability to function as nanochannels for fluids^32^ and templates for nanomaterial fabrications with novel properties^33^. The rapid transport of liquids through carbon-shell channels due to non-wettability has been widely reported^34^,^35^,^36^. In contrast, metals have been encapsulated into carbon-shell confinements to produce novel composites with potential applications in semiconductor, storage and nanomagnetic devices^37^ using various techniques^38^,^39^. Besides, wetting and coalescence inevitably occur when a liquid interacts with CNT or G. Therefore, understanding these two processes plays an important role in fabrications of materials.

However, there are few studies concerning the wettability and coalescence of metallic droplets in two-wall confinement. Moreover, tunable wettability and the mobility of liquid fillers have emerged from the perspective of large-scale industrial applications of droplets in nanodevices^40^, although numerous efforts have been exerted on unexpected confinement-induced structures and the properties of fillers^41^,^42^,^43^. Therefore, we performed simulations concerning the wettability and movement of Cu droplets confined in two carbon walls composed of double-wall graphene (DG), horizontal or vertical carbon nanotubes (HCNTs or VCNTs), to probe the effectively controllable droplet behavior by detachment and confinement.

## Results

### One drop in confinement

**Figure 1:**
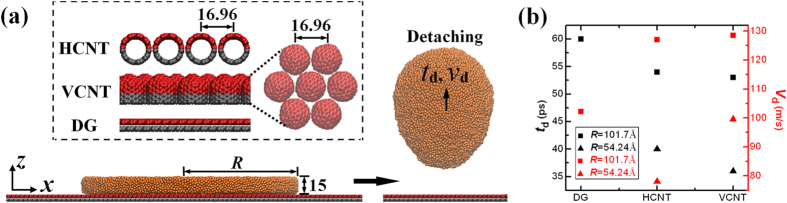
Liquid dynamics of Cu droplets on carbon walls. (**a**) Detachment of a Cu droplet. The drop detaches at a certain time. Three types of carbon walls are considered: DG, HCNT and VCNT. The CNTs with a diameter of 13.56 Å are 16.96 Å away from their neighbors, with an interval distance of 3.4 Å. HCNT is composed of uncapped CNTs and VCNT is composed of capped CNTs. All of the walls are deposited in the *xoy* plane, with the part (red atoms) near the Cu film fixed at the same temperature as the Cu films and the remaining part (gray atoms) remaining frozen. (**b**) The corresponding detaching times (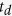) and speeds (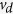), which are highly related to the types of walls and the radius of the liquid film.

**Figure 2:**
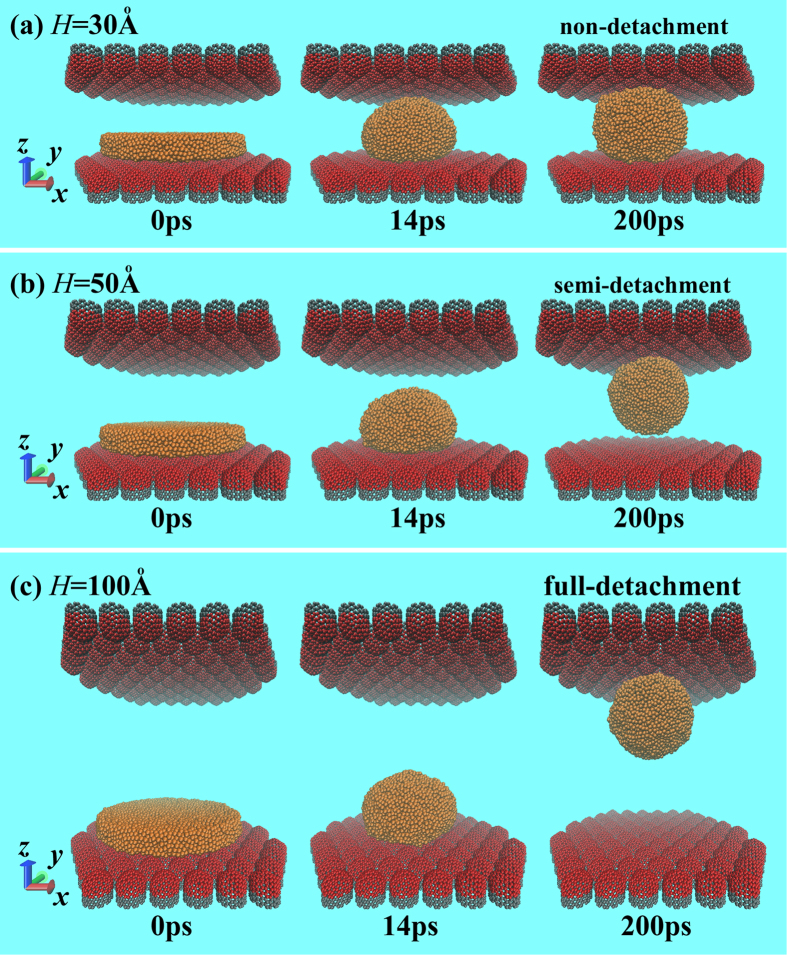
Snapshots of liquid dynamics in the VCNT confinement. The droplets finally display three states, as follows: non-detachment (**a**), semi-detachment (**b**) and full-detachment (**c**), depending on the heights (*H*) of the confinement. This reveals that the controllable formation behavior of the droplet is available via tuning the *H*.

**Figure 3:**
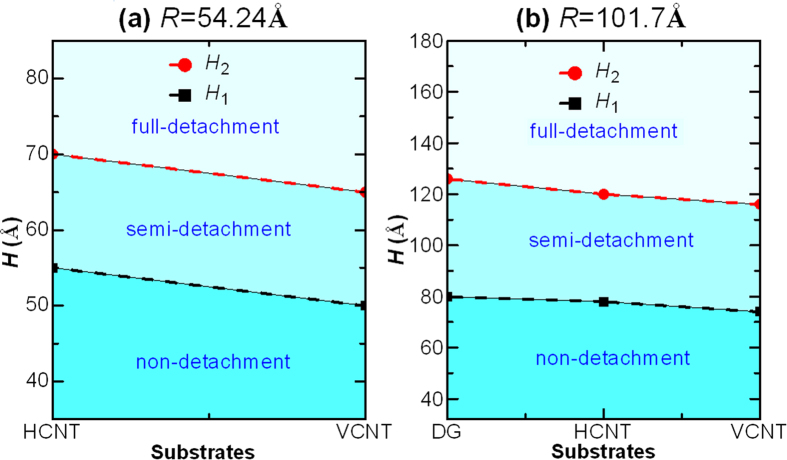
The critical heights, *H*_1_ and *H*_2_, between the three states for systems *R* = 54.24 and 101.7 Å, respectively. The final droplet displays the non-detachment state in the region below the black line, the full-detachment state in the region above the red line and the semi-detachment state in the region between the two lines.

Figure 1 shows that liquid Cu films with a large surface-volume ratio on the carbon wall can detach from the wall due to dewettability, with detaching time (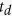) and speed (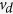) over wide ranges, which are significantly related to the geometric parameters of the liquid films, as well as the surface structures of the carbon walls. What would occur if the detached drop impacts another wall with the same wettability as the bottom one? Figure 2 presents the dynamic snapshots of one Cu liquid film (*R* = 54.24 Å) in the two VCNT-walls. Once placed on the wall, *i.e.,* the bottom wall, the film contracts rapidly to form a droplet. This contracted droplet, however, displays different states in the confined space with different heights (*H*). For example, the contracted drop exhibits a non-detachment state when *H* = 30 Å, a semi-detachment state when *H* = 50 Å or a full-detachment state when *H* = 100 Å. In the first state (Fig. 2a), the droplet contacts the top wall during the contracting process. Due to the limited space, the contracted droplet is unable to jump off the bottom wall, finally displaying a drum-like shape and a strong interaction with the bottom wall, which suggests that the droplet contacts with the bottom wall on a surface. In the second state (Fig. 2b), the contracted droplet displays a completely different shape in which it is spherical and appears to have detached from the bottom wall. However, there exists a weak attractive force between the drop and the bottom wall. That is, the droplet contacts with the bottom wall at a point, indicating that the detachment is incomplete. Therefore, this state is defined as a semi-detachment state. In the last state (Fig. 3c), the contracted droplet fully detaches form the bottom wall at a certain time and collides with the top wall without any interaction with the bottom wall. The full-detachment process had been used by Boneberg *et al.*^30^ to collect droplets. Additionally, Boneberg *et al.*^30^ found that the collected particle of a small size was in a spherical shape, while the largest particle, which required the longest time to cool, exhibited a spherical calotte shape. Thus, the collected droplets of different sizes finally displayed different shapes due to the different cooling rates. At this stage, if the time permits, the impacting droplets finally display spherical shapes similar to that in the semi-detachment state due to the constant temperature of the droplet. To qualitatively determine the critical heights among these states, a series of MD simulations are performed on different systems, *i.e.,* different Cu films (*R* = 54.24 and 101.7 Å), different carbon walls (DG, VCNT and HCNT) and different confined spaces (*H* ranging from 10 to ~240 Å). Figure 3 shows that the contracted droplet is in the non-detachment state when *H* ≤ 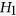, in the full-detachment state when *H* ≥ 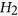 and in the semi-detachment state when *H* is between 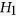 and 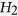, indicating that the states of the liquid droplet can be precisely controlled by tailoring the height of the confined space. In Habenicht *et al.*^3^ and Boneberg *et al.*^30^, the non-detachment (full detachment) state was obtained by decreasing (increasing) the laser energy density. However, a discussion on the semi-detachment state was missing from these studies, possibly because of the difficulty in precisely controlling the laser energy density to obtain the semi-detachment state.

**Figure 4:**
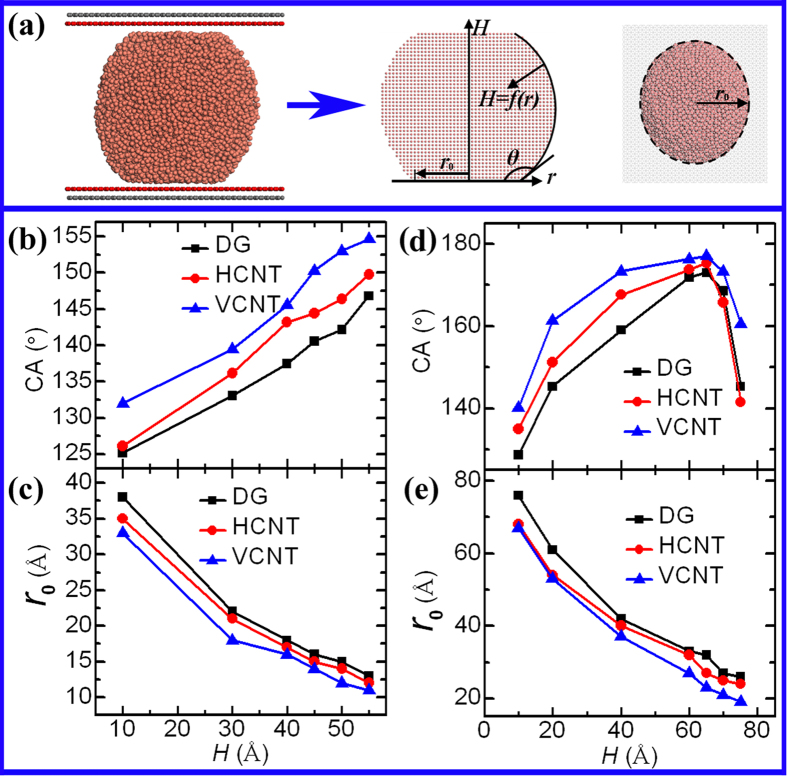
The wettability of the Cu droplet in the non-detachment state. (**a**) Measurements of the contact angle (CA, 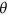) and radius (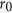) of the liquid-solid contact area. First, the drum-like drop is meshed into grids with a length of 2 Å, followed by the calculation of the atomic density of each grid. Second, the surface function, 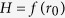, of the drop is depicted and is fitted by the polynomial functions. Finally, the CA and 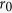 are extracted from the drop’s contour line 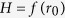. The CA is obtained at the equilibrium by averaging the instantaneous CAs on time steps 410, 420, 430, …, 480, 490, 500 ps. (**b–e)** The two key parameters (CA and 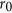) as functions of *H* for the systems *R* = 54.24 and 101.7 Å, respectively.

The contact angle (CA) and radius (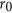) of the contact area are valuable for analyzing the wettability of droplets at the non-detachment state. Figure 4a illustrates the measurement of the CA. For the systems *R* = 54.24 Å (Fig. 4b,c), the CA dramatically increases, and the contact area at the liquid-solid interface rapidly declines as *H* increases. For example, in the VCNT-confinement, at *H* = 10 Å, the CA and 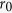 are 132.02° and 33 Å, respectively, whereas at *H* = 55 Å, the CA and 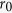 are 154.68° and 11 Å, respectively, which suggests that there is a close relationship between the wettability and the height of the confined space. This relationship, however, becomes relatively complicated in the systems in which *R* = 101.7 Å. As shown in Fig. 4d, the CA rapidly increases nearly to 180° at the point where *H* = 65 Å, but after this point, it declines at a faster rate, and finally diminishes to the value obtained in free space, implying the important role of the droplet size in determining the CA. Koishi *et al.*^44^ had reported that the contact angle displays a monotonously increasing trend when the number of molecules varies from 1000 to 22000. In our case the number of Cu atoms varies from 10120 to 34992, yet the increasing tendency of the CA is observed. Notably, for the same value of *H*, the VCNT confinement in both of the systems (*R* = 54.24 and 101.7 Å) possesses the largest CA and the lowest 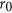, followed by the HCNT confinement, which indicates that the surface structures of the carbon walls play a crucial role in wettability^26^. Moreover, the CA and the contact area in all of the systems vary over a wide range with the various *H*. For example, the CA in the DG-confinement (*R* = 101.7 Å) increases from 128.72° to 172.99°, and the corresponding 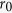 decreases from 76 to 26 Å as *H* increases from 10 to 65 Å, revealing that regulation of the confined space is an effective method to control liquid dynamics, regardless of the initial conditions.

**Figure 5:**
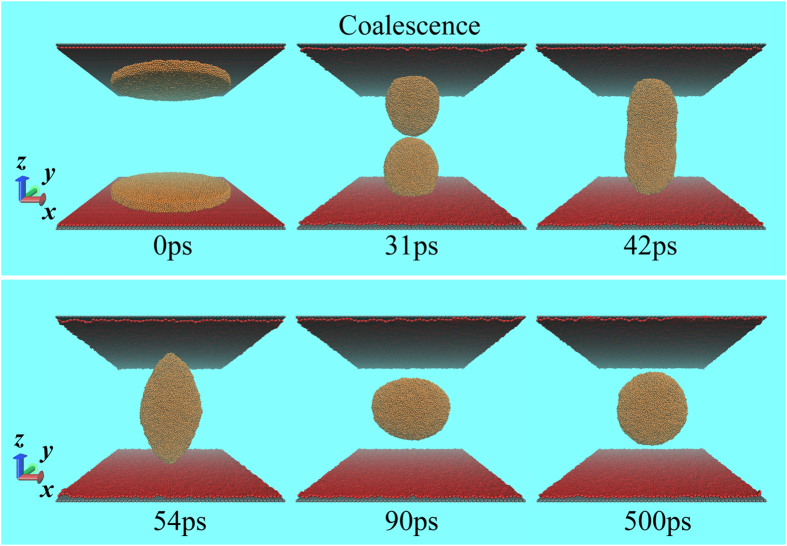
The spontaneously coalescing process in the DG confinement (*H* = 160 Å). The two films (*R* = 101.7 Å) that are initially distant from each other coalesce into one larger drop. The merged drop is finally located at the middle position of the confinement.

The dependence of *H* on the movement of the detached droplet in the full-detachment state as shown in Figure S1 (see the Supplementary Materials). For the systems in which *R* = 54.24 Å (Figure S1a), 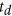 remains constant, whereas the contact time (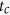) increases nearly linearly with the increasing *H*, leading to linear increases of the interval times (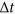), which are defined as the moving time of the ejected droplet, as indicated by the dashed line. These findings resemble those found in the systems in which *R* = 101.7 Å (Figure S1b). Habenicht *et al.*^3^ reported that a larger laser capacity indicates a faster jumping speed of the droplet. Fuentes-Cabrera *et al*^22^ demonstrated that a higher temperature causes a faster detaching speed of the droplets. Therefore, the 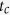 and 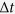 are markedly lower in the systems with larger radius (*R* = 101.7 Å) of the liquid film because the detaching speed in this case is larger than that in the case of *R* = 54.24 Å. Thus, the larger the *R*, the faster the detaching speed and the shorter the 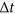. Additionally, Fig. 5c shows that the VCNT-confinement in every system possesses the smallest slope of the 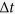-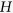 line, and in every confinement, the slope is markedly smaller when *R* = 101.7 Å than when*R* = 54.24 Å. Thus, tuning the *H* leads to dramatic changes in 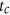 and 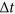 rather than 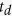. Overall, this analysis indicates that the movement of the contracted droplet is sensitive to the initial conditions of the system.

### Two drops in confinement

Figure 5 shows snapshots of the spontaneous coalescence of two droplets in the two DG walls (*H* = 160 Å). The two liquid films (*R* = 101.7 Å) are separately deposited on each wall. At the initial stage, each film rapidly shrinks into a droplet, while gradually approaching each other due to the velocity component in the z-direction. As the simulation proceeds, the two drops come in contact and instantly merge to form a larger drop, which is finally located at the middle position of the confinement. Similar phenomenon is also found in the HCNT and VCNT confinements, indicating that the two droplets in the two-wall confinement can spontaneously coalesce via detachment, regardless of the long distance initially between them. By employing a similar two-wall confinement, Yaneva *et al.*^45^ obtained reverse results in which the continuous polymer film gradually ruptured with the increasing height of the confined space because the walls contained lyophilic stripes. Thus, the liquid dynamics in this type of confinement, such as rupture and coalescence, can be controlled by tuning the wettability of the two walls.

**Figure 6:**
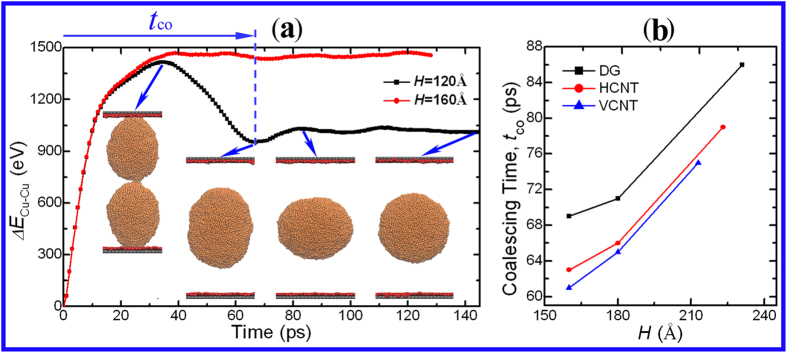
(**a**) The unexpectedly spontaneous merging process of the drops in the DG confinement (*R* = 54.24 Å and *H* = 120 Å). This behavior is not observed when *H* = 160 Å. 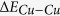 is the different potential energy of the Cu atoms, defined as 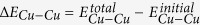. The 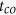 is defined as the coalescing time from the beginning to the time when the 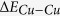 reaches the lowest value after the drops come into contact. (**b**) The coalescing time *versus H* for the system *R* = 101.7 Å.

Unexpectedly, the spontaneous coalescence is also found in the DG-confinement (*R* = 54.24 Å), given the condition that *H* varies from 80 to 120 Å. Figure 6a shows that the contracted drops detach from the walls and coalesce after they come into contact, which is not found when *H* is sufficiently large. A better understanding of this phenomenon is gained by the comparison of energy-*t* curves between the cases in which *H* = 120 and 160 Å. In the latter case, the contracted droplets continue to adhere to the walls without detachment and contact, resulting in non-coalescence. The energy continues to increase until the system reaches the equilibrium. However, in the case in which *H* = 120 Å, the energy increases to the maximum at the point (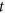 = 36 ps) where the contracted drops initially make contact; however, after this point, it rapidly decreases to a minimum at the point (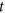 = 66 ps) where the merged drop is in the spherical shape. The dramatic decrease of the energy reveals that the coalescence is spontaneous. Figure 6b plots the coalescing time (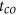) as a function of *H* to gain insight into the coalescence. Clearly, the coalescing time (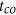) dramatically increases with the increasing *H*. Notably, at every value of *H*, the VCNT confinement possesses the lowest 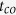, which is markedly lower than that in the DG confinement. Moreover, 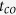 ranges over 100 ps, implying the rapid spontaneous coalescence of droplets in the confinement.

**Figure 7:**
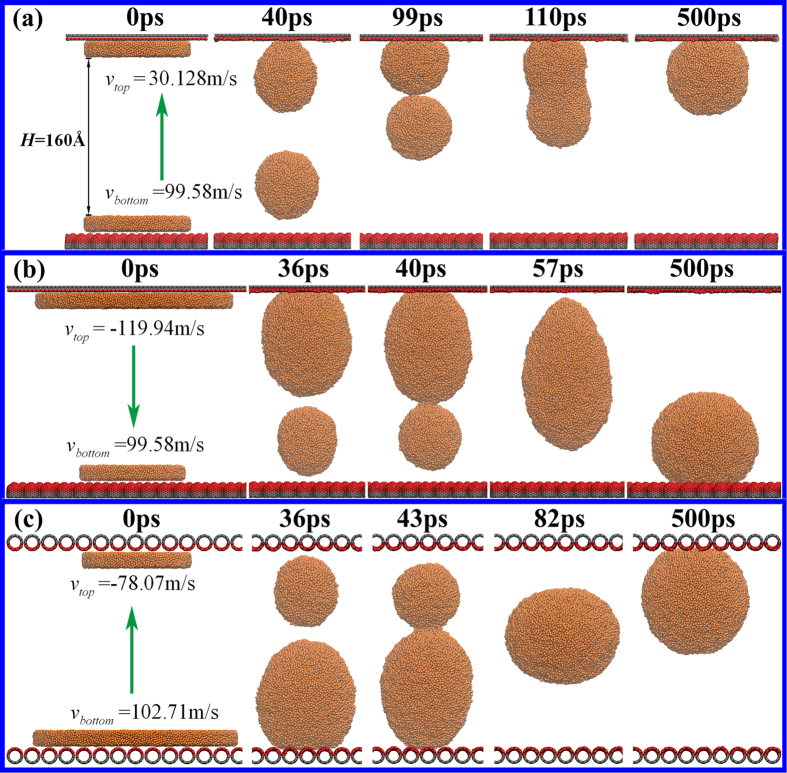
Snapshots of the coalescing process. The merged drop finally adheres to the top wall (**a**) and (**c**) or is located on the bottom wall (**b**). Parameters 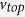 and 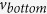 are the velocities of the droplets at the contact moment. The negative velocity represents the movement along the direction opposite to the z-direction. The coalesced drop moves along the direction labeled by the green arrows.

The final position of the coalesced drop is another important feature that requires consideration. In Fig. 6, the merged drop is finally located at the middle position of the symmetrical confinement, which is different from the previous results. For example, the jumping droplet landed on the top wall in the study by Boneberg *et al.*^30^, and the polymer film ruptured into two separate drops that landed separately on the two walls in the study by Yaneva *et al.*^45^. Figure 7 shows that the final position can be varied by changing the geometric parameters of the liquid film, the surface structures of the carbon walls or both of these features. In the case of the same *R* but with two different walls (Fig. 7a), the bottom drop initially jumps off the bottom wall (VCNT), and then gradually approaches the other drop, followed by the full coalescence process. Finally, the merged drop adheres to the top wall (DG). The final position of the droplet would be opposite, provided that the *R* of the top film increases from 54.24 to 101.7 Å. As shown in Fig. 7b, the fully coalesced drop finally adheres to the bottom wall. Thus, the merged drop tends to move to the position where the smaller drop was initially deposited. Similar phenomenon is also found in the case with the same walls but with different *R* (Fig. 7c), which indicates that the movement of the larger drop plays a dominant role in determining the final position of the merged drop. To verify this idea, the velocities of the two drops at the moment of contact are calculated. The results show that the moving direction of the coalesced drop depends on the direction of the droplet with the faster velocity, suggesting that the merged droplet is finally located on the surface that displays a relatively weak dewettability. That is, the merged droplet moves to the top (bottom) wall if the liquid film on this wall possessing a smaller detaching velocity than the bottom (top) one. Therefore, it can be concluded from Fig. 7 that the final position of the coalesced drop in the two-wall confinement can be effectively controlled by tuning the initial conditions of the system, *i.e.,* the geometric parameters of the liquid film and the surface structures of the carbon walls.

## Discussion

**Figure 8:**
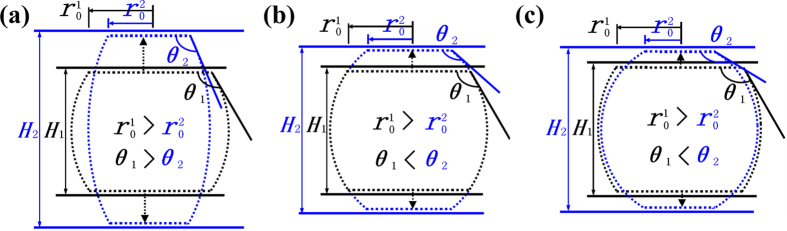
The wetting mode in the non-detachment state. The black and blue dot lines represent the drop shapes at two random moments with the confined space of 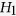 and 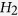, respectively. (**a**) The drop is elongated with its surface curvature decreasing, resulting in the decrease of the CA, which contradicts the results in Fig. 4. (**b**) The drop is elongated without changes in its surface curvature, leading to the increase of the CA, which is consistent with the results in Fig. 4; however, this wetting mode contradicts the law in which the drop volume remains constant. (**c**) The drop shape varies with the increase of the drop curvature and the rapid decrease of the contact area, with both of the factors acting together to induce the rapid increase of the CA. The results in Fig. 4 can be well explained by this theoretical model.

The CA is expected to decrease with increasing *H* because the elongation of the drop may lead to the decrease of its curvature, as indicated in Fig. 8a. Unexpectedly, the CA dramatically increases as *H* increases (Fig. 4). In Fig. 8b, we hypothesis that the curvature of the droplet remains constant when *H* increases. Based on this hypothesis, the CA increases and the 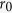 decreases with the increasing *H*. That is, the results in Fig. 4 can be well explained by this hypothesis. However, the volume of the droplet must increase, which contradicts the law that the volume of the droplet is constant. Therefore, the increase of surface curvature and the decrease of contact area result from the increase of *H* (Fig. 8c). Moreover, the results from Fig. 4 imply the dominant role of the latter factor in the manipulated wettability.

Due to the contraction of the liquid film, the relationship between 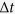 and *H* in the full-detachment state can be depicted as 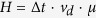 by introducing the modifying factor 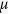. As shown in Table S1 (see the Supplementary Materials), the magnitude of 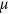 remains practically constant, regardless of the initial conditions of the system. Thus, the controllability of the ejected droplet is not subject to the noise, and the carbon wall type can affect the relationship between the moving time and the height of the confined space. Thus, the dynamics of the detaching droplet can be precisely controlled by tuning the confined space, based on the given 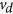.

In Huang *et al.*^31^ and in Afkhami and Kondic^23^, an Au droplet displayed similar dewettability to Cu. Hence, based on the liquid dynamics analysis of Cu droplets, including 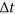, 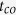 and coalescing position, similar confinement effects would be observed in Au. The 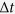 will be accurately controlled by tuning the *H* according to the linear relationship between 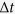 and *H*. Simultaneously, the spontaneous coalescence is expected to occur due to the detachment, regardless of the initial distance between the two drops. Furthermore, the coalescing position can be predicted by properly regulating the initial conditions of the system.

In summary, an MD simulation was performed to study the droplet dynamics of un-contacted Cu films in two carbon walls, including the wettability and spontaneous coalescence induced by dewettability, which are sensitive to the geometric parameters of the liquid film, the surface structures of the carbon walls and the height of the confined space. This study reveals the possibility of droplet dynamics control (including wettability and coalescence) and promising applications of spontaneous coalescence in uniform metal droplet spray^46^, ink-jet printing^47^,^48^ and droplet reactors^49^.

## Methods

The circular Cu films with a thickness of 15 Å but with different radii (*R* = 54.24 and 101.7 Å), are extracted from a bulk liquid at 1500 K and placed at a distance of 3.225 Å away from the carbon walls. The Cu film contains 34992 or 10120 atoms when *R* = 54.24 or 101.7 Å, respectively. The two-wall confinement is constructed of two mutually parallel carbon walls. In this study, three types of carbon walls are considered, as shown in the inset in Fig. 1. The CNT wall consists of single-wall CNTs (10,10) with an interval distance of 16.96 Å. To avoid interaction between Cu and C at high temperatures, the VCNT wall is composed of capped CNTs (10, 10) with the caps facing the liquid film. The distance between the boundaries of the liquid film and the substrate exceeds 20 Å to avoid the boundary effect. For all of the substrates, the bottom parts (labeled in gray) are fixed, and the top parts (marked in red) remain at 1500 K as Cu films. Cu-Cu interactions are described by an embedded atom method (EAM) potential^50^. Interactions among C atoms are modeled by an adaptive intermolecular reactive empirical bond order (AIREBO) potential^51^. The Lennard-Jones (LJ) potential with well depth parameters *ε* = 0.01 eV and *σ* = 3.225 Å is utilized to calculate the Cu-C interactions^23^,^52^. This LJ potential has been widely determined on the Cu-C system with the equilibrium contact angle (CA = 133°, which is an average measured when a droplet on the DG reaches the equilibrium) similar to the theoretical and experimental results^22^,^53^. Molecular dynamics (MD) simulations are performed at constant volume and temperature with the large-scale atomic/molecular massively parallel simulator (LAMMPS) package^54^,^55^. The initial configuration is at rest, and the system is coupled to the Nosé−Hoover thermostat^56^,^57^ with a time constant of 0.5 ps^58^. The time integration of Newton’s equation of motion is undertaken using the velocity Verlet algorithm^59^. The time step is 1.0 fs and all of the MD simulations are run for 500 ps.

## Additional Information

**How to cite this article**: Li, X. *et al.* Wettability and coalescence of Cu droplets subjected to two-wall confinement. *Sci. Rep.* **5**, 15190; doi: 10.1038/srep15190 (2015).

## Supplementary Material

Supplementary Information

Supplementary Video 1

Supplementary Video 2

Supplementary Video 3

Supplementary Video 4

Supplementary Video 5
