# Supplementary material for: Wettability and Coalescence of Cu Droplets Subjected to Two-Wall Confinement
Source: Sci Rep. 2015 Oct 13;5:15190. doi: 10.1038/srep15190 (PMC4602311; doi:10.1038/srep15190)
Supplement: Supplementary Information [file srep15190-s1.doc]

**Wettability and Coalescence of Cu Droplets Subjected to Two-Wall Confinement**

Xiongying Li^1^, Hongru Ren^1^, Weikang Wu^1^, Hui Li^1^,*, Long Wang^1^, Yezeng He^2^, Junjun Wang^1^ & Yi Zhou^1^

^1^*Key Laboratory for Liquid-Solid Structural Evolution and Processing of Materials, Ministry of Education, Shandong University, Jinan 250061, People’s Republic of China*

^2^*School of Material Science and Engineering, China University of Mining and Technology, Xuzhou 221116, P. R. China*

**lihuilmy@hotmail.com*

**Supplementary Information**

**PS1: The movement of one Cu droplet in the full-detachment state**

The moving time of the droplet, defined as $\Delta t=t_{\text{c}}-t_{\text{d}}$ ( $t_{\text{c}}$ is the time when the drop contacts the top substrate), shows a linear relationship with $H$, as indicated by the dashed lines. The corresponding slope has a decreasing tendency as the walls change from DG to HCNT then to VCNT or as the $R$ decreases.


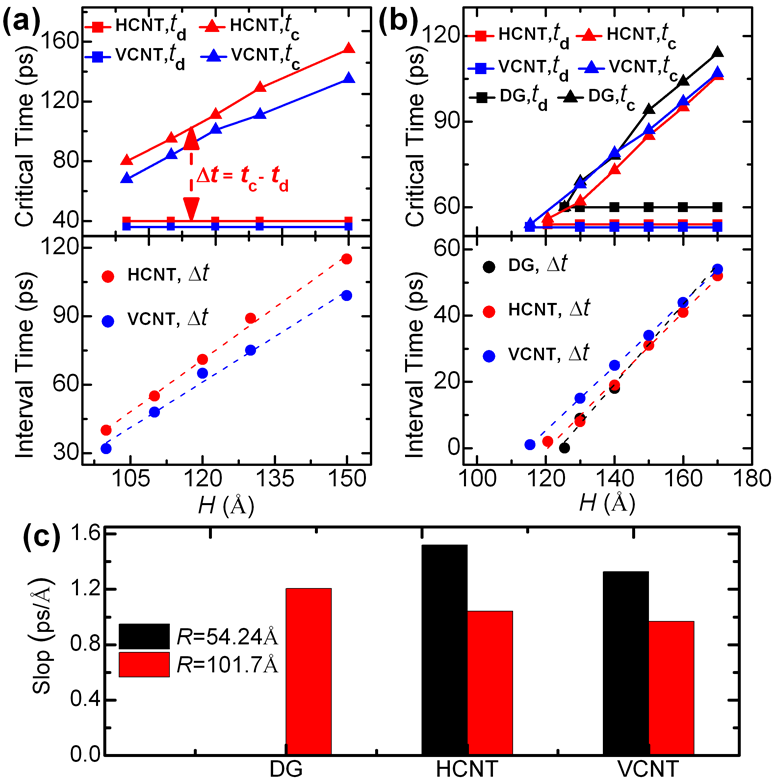


**Figure S1**. Liquid dynamics in the full-detachment state. (a) and (b) Moving times ($\Delta t=t_{c}-t_{d}$) for the systems $R$=54.24 and 101.7 Å, respectively. (c) The slope of the dashed line.

**PS2: The relation between $H$ and** $\Delta t$

**Table S1**. The modifying factor ($\mu$) in the function $H=\Delta t\cdot v_{d}\cdot\mu$

| **Substrates** | ***R*=54.24Å** | | ***R*=101.7Å** | | |
| --- | --- | --- | --- | --- | --- |
|  | **HCNT** | **VCNT** | **DG** | **HCNT** | **VCNT** |
| $\mu$ | **1.187** | **1.320** | **1.232** | **1.322** | **1.245** |

**PS3: Video legends**

**Video1**: Dynamics of one Cu film ($R$=54.24 Å) in the HCNT-confinement (*H*=30 Å).

**Video 2**: Detaching dynamics of one Cu film ($R$=54.24 Å) in the HCNT-confinement (*H*=100 Å).

**Video 3**: Spontaneous coalescence of two Cu films ($R$=101.7 Å) in the VCNT-confinement (*H*=160 Å).

**Video 4**: Spontaneous coalescence of two Cu films ($R$=54.24 and 101.7 Å, respectively) in the VCNT-confinement (*H*=160 Å).

**Video 5**: Spontaneous coalescence of two Cu films ($R$=54.24 and 101.7 Å, respectively) in the confinement (*H*=160 Å) made of one VCNT wall and one HCNT wall.
